# Analysis of 24-hour Death Risk Factors in Circulatory Failure Patients Treated with Venoarterial Extracorporeal Membrane Oxygenation

**DOI:** 10.21470/1678-9741-2022-0398

**Published:** 2023-09-11

**Authors:** Jianwei Wang, Shengshu Wang, Yang Song, MingJun Huang, Wenzhe Cao, Shaohua Liu, Shimin Chen, Xuehang Li, Miao Liu, Yao He

**Affiliations:** 1 Institute of Geriatrics, Beijing Key Laboratory of Aging and Geriatrics, National Clinical Research Center for Geriatrics Diseases, Second Medical Center, Chinese People's Liberation Army General Hospital, Beijing, People’s Republic of China; 2 Chinese People’s Liberation Army Medical School, Beijing, People’s Republic of China; 3 Department of Critical Care Medicine, Puyang People’s Hospital, Henan, People’s Republic of China; 4 Department of Extracorporeal Support Center, The First Affiliated Hospital of Zhengzhou University, Zhengzhou, People’s Republic of China; 5 Department of Statistics and Epidemiology, Graduate School, Chinese People’s Liberation Army General Hospital, Beijing, People’s Republic of China; 6 State Key Laboratory of Kidney Diseases, Beijing, People’s Republic of China

**Keywords:** Extracorporeal Membrane Oxygenation, Prognosis, Risk Factors, Cardiopulmonary Failure, Hospital Mortality

## Abstract

**Objective:**

To explore the factors affecting short-term prognosis of circulatory failure patients undergoing venoarterial extracorporeal membrane oxygenation (VA-ECMO) treatment.

**Methods:**

A total of 136 patients undergoing VA-ECMO were enrolled in this study and subsequently divided into the death group (n=35) and the survival group (n=101) based on whether death occurred during hospitalisation. Extracorporeal membrane oxygenation (ECMO) running time, length of intensive care unit stay, length of hospital stay, costs, and ECMO complications were then compared between the two groups.

**Results:**

The average age of all patients undergoing ECMO was 47.64±16.78 years (53.2±16.20 years in the death group and 45.713±16.62 years in the survival group) (P=0.022). Patients in the survival group exhibited a clear downward trend in lactic acid value following ECMO treatment compared to those in the death group. Total hospitalisation stay was longer in the survival group (35 days) than in the death group (15.5 days) (P<0.001). In the analysis of ECMO complications, the incidence of neurological complications, renal failure, limb complications, and infection were higher in the death group than in the survival group (P<0.05 for all). Specifically, as a risk factor for patient survival and discharge, the occurrence of infection will lead to increased hospitalisation stays and costs (P<0.05 for both).

**Conclusion:**

Complications such as kidney failure and infection are associated with in-hospital death, and ECMO-related complications should be actively prevented to improve the survival rate of VA-ECMO treatment.

## INTRODUCTION

**Table:** 

Abbreviations, Acronyms & Symbols	
BMI	= Body mass index
CPR	= Cardiopulmonary resuscitation
CRRT	= Continuous renal replacement therapy
ECMO	= Extracorporeal membrane oxygenation
ECPR	= External cardiopulmonary resuscitation
ELSO	= Extracorporeal Life Support Organization
IABP	= Intra-aortic balloon pump
ICU	= Intensive care unit
Lac	= Blood lactate
OR	= Odds ratio
VA-ECMO	= Venoarterial extracorporeal membrane oxygenation

Venoarterial extracorporeal membrane oxygenation (VA-ECMO) is a mediumand short-term cardiopulmonary mechanical auxiliary technique that involves draining venous blood *in vitro* through artificial pipelines^[1]^. After excluding the carbon dioxide via oxygen exchange, the oxygenated arterial blood is repumped into the patient by the driving pump^[2]^. Treatment with extracorporeal membrane oxygenation (ECMO) can provide effective circulatory respiratory support for patients with critical cardiopulmonary failure caused by various factors, stabilising the patient’s basic vital signs, an effective mean of saving patients with critical diseases^[3,4]^. With the development of ECMO technology, the treatment can be performed at the bedside, which is beneficial for patients with a severe condition experiencing hemodynamic instability and being unsuitable for transference, meaning the use of ECMO has become increasingly widespread in clinical settings^[5]^. The ECMO technique outperforms other circulatory techniques, such as those using intra-aortic balloon counter-pacing devices^[6-8]^, the TandemHeart® left ventricular assist^[9]^, and the Impella® device^[10]^. The advantages of ECMO include the rapid establishment of circulation, supporting right ventricular, left ventricular, or biventricular failure at high blood flow rates, and providing supportive treatment for patients with lung damage^[3,11]^.

However, while this technology provides significant treatment benefits, patients with a severe disease are still prone to adverse events and even death in the hospital. Early implementation of targeted interventions determined according to the risk factors of patients undergoing VA-ECMO will help to improve their prognosis. However, there remains some uncertainty surrounding which factors could influence the survival of critical patients who receive VA-ECMO treatment. Therefore, this study aimed to explore the factors affecting the short-term (24-hour) prognosis of critical patients undergoing VA-ECMO in a retrospective cohort, providing a basis for further improving the survival rate among such patients.

## METHODS

### Study Design

This single-centre retrospective study was approved by the Ethical Committee of the Chinese PLA General Hospital (2021-E-03-001) and conforms to the STrengthening the Reporting of OBservational studies in Epidemiology (or STROBE) statement. All methods were performed in accordance with the relevant guidelines and regulations. Family members of the patients signed the informed consent form.

### Study Population

The cohort included 136 patients who received VA-ECMO treatment and survived beyond the 24-hour period following the treatment in the Chinese PLA General Hospital between June 2016 and October 2019. The patients were divided into two groups: the death group (died during hospitalisation, n=35) and the survival group (survived to discharge, n=101).

The inclusion criteria were as follows: (1) patients aged between 18 and 75 years; (2) patients diagnosed with emergency refractory cardiac shock or cardiac arrest and requiring VA-ECMO assistance (refractory cardiogenic shock refers to hypotension that is difficult to control via conventional treatment and insufficient perfusion; heart insufficiency can be detected via echocardiography, while refractory cardiac arrest refers to failing to recover or maintain spontaneous circulation following 10 minutes of traditional cardiopulmonary resuscitation [CPR]); and (3) patients who survived beyond 24 hours following ECMO withdrawal.

The exclusion criteria were as follows: (1) patients with end-stage malignancies or significant coagulation dysfunction; (2) patients with severe chronic disease affecting prognosis; (3) patients with incomplete data; and (4) patients who were withdrawn due to financial problems.

The indications for ECMO were as follows: (1) the extracorporeal circulation machine was directly transferred to ECMO adjuvant support without being interrupted; (2) the low cardiac output (cardiac index < 2.0 L/min·m^2^) was difficult to correct; (3) occurrence of severe hypoxemia; or (4) occurrence of cardiac arrest. These diagnostic criteria were determined with reference to the consensus-based guideline (level S3) for extracorporeal life support/ECMO therapy, which was developed under the regulations of the Association of the Scientific Medical Societies in Germany and the Grading of Recommendations Assessment, Development and Evaluation criteria^[9]^.

In terms of ECMO weaning criteria, following the recovery of the patient’s cardiac function and other organ functioning, the flow rate was gradually reduced to 1-1.5 L/min. Then, after reducing the flow for one hour, the indicators (echocardiographic ejection fraction ≥ 25%-35%, no or small-dose vasoactive drugs, pulse pressure difference ≥ 30 mmHg, and no blood lactate [Lac] elevation) were assessed to determine whether withdrawal was appropriate. During withdrawal, the femoral artery catheter was removed using the method of vascular surgical artery thrombolysis and rupture repair, while for the femoral vein catheter, the method of compression and stopping the bleeding was adopted.

### Data Collection

The patients’ demographic and clinical diagnosis data were obtained from the corresponding electronic medical records, with the indications, canulation technical characteristics, and clinical events pertaining to ECMO recorded. The main data included ECMO running time, intensive care unit (ICU) stay, hospital stay and costs, and ECMO-related complications.

### Statistical Analysis

The data were analysed using IBM Corp. Released 2019, IBM SPSS Statistics for Windows, version 26, Armonk, NY: IBM Corp. In terms of continuous variables, the normality of the distribution was assessed using the Kolmogorov-Smirnov test, with all measurement data conforming to the normal distribution expressed as mean ± standard deviation. Inter-group comparisons were performed using two independent sample *t*-tests. Measurement data that did not follow the normal distribution were expressed in terms of the median (interquartile range; M: P25, P75), with the Mann-Whitney U test used for inter-group comparisons. The count data were expressed in terms of number (n) and percentage (%), with the Chi-square test used for inter-group comparisons and Fisher’s exact probability method used where appropriate. Risk factors associated with the clinical prognosis were analysed via binary logistics regression. A two-tailed *P*-value of < 0.05 was considered to be statistically significant.

## RESULTS

### Demographic Characteristics

A retrospective analysis of the baseline characteristics indicated that the average age of all the patients receiving ECMO implantation was 47.64±16.78 years, with 53.2±16.20 years for the death group and 45.713±16.62 years for the survival group; the difference between the two groups was statistically significant (*P*=0.022). The recruited patients were predominantly male (66.2%), while there was no statistically significant difference in terms of gender, body mass index, previous history of diabetes, hypertension, cerebrovascular accident, and cardiac failure, or inter-hospital transport rate between the two groups. There were no statistically significant differences in terms of whether the patients underwent CPR due to cardiac arrest or in the worst Lac value prior to ECMO treatment between the two groups. After 24 hours of ECMO treatment, the patients in the survival group (2.2 [1.2, 3.125]) exhibited a better downward trend in Lac than those in the death group (2.4 [1.9, 4.8]) (*P*=0.024). There was no statistically significant difference in terms of ECMO running time or ICU stay between the two groups, while the total hospitalisation time was longer in the survival group (35 [20, 47.75] days) than in the death group (15.5 [11.5, 24] days) (*P*<0.001). There was no statistical difference in the total hospitalisation cost between the two groups (Table 1).

**Table 1:** Comparison of demographic and clinical characteristics.

	Total (n = 136)	Death group (n = 35)	Survival group (n = 101)	t/χ2	*P*-value
Age (years)	47.64 ± 16.779	53.2 ± 16.2	45.713 ± 16.622	2.311	0.022
Male	66.2% (90)	71.4% (25)	64.4% (65)	-0.759	0.448
BMI	23.968 ± 3.704	24.257 ± 3.851	23.862 ± 3.663	0.538	0.592
Diabetes	14% (19)	11.4% (4)	14.9% (15)	0.049	0.825
Hypertension	25.7% (35)	31.4% (11)	23.8% (24)	0.799	0.371
Cerebrovascular accident	5.9% (8)	11.4% (4)	4% (4)	1.443^a^	0.23
History of heart failure	18.4% (25)	17.1% (6)	18.8% (19)	0.048	0.826
ECMO inter-hospital transport	21.3% (29)	20% (7)	21.8% (22)	0.049	0.824
CPR before ECMO use	23.5% (32)	22.9% (8)	23.8% (24)	0.012	0.913
Worst Lac value before ECMO use	6.85 (2.375, 12.225)	7.4 (2.95, 13.75)	6.4 (2.15, 12.05)	-0.394	0.694
Lac value 24 hours after ECMO	2.3 (1.3, 3.4)	2.4 (1.9, 4.8)	2.2 (1.2, 3.125)	-2.251	0.024
ECMO running time (hours)	85.5 (44.5, 124.5)	108 (60, 140.6)	83.6 (39.2, 119.2)	-1.828	0.068
ICU stay (days)	14 (9, 23)	13 (9, 18.25)	14 (8.7, 23.25)	-0.541	0.588
Hospital stay (days)	29 (16, 43.75)	15.5 (11.5, 24)	35 (20, 47.75)	-4.189	< 0.001
Hospital cost (Ten thousand Yuan)	35.6 (25.25, 53.825)	42.55 (32.95, 53.3)	32.65 (22.85, 53.875)	-1.855	0.064

BMI=body mass index; CPR=cardiopulmonary resuscitation; ECMO=extracorporeal membrane oxygenation; ICU=intensive care unit; Lac=blood lactate

a The difference was conducted by Fisher’s exact chi-square test

### Indications and Technical Characteristics of Extracorporeal Membrane Oxygenation

During the establishment of VA-ECMO, there were no statistical differences in ECMO indications either in the patients with circulatory failure alone, circulatory failure combined with respiratory failure or respiratory failure alone (patients undergoing lung transplantation), or in those with external CPR. There was also no significant difference in the VA-ECMO establishment sites, which included operating room, monitoring room, emergency room, and catheter room. The difference was not statistically significant between the two groups in terms of ECMO combined with intra-aortic balloon pump treatment (Table 2).

**Table 2:** Indications and technical characteristics of ECMO.

	Total (n = 136)	Death group (n = 35)	Survival group (n = 101)	χ2	*P*-value
Classification of ECMO indications				1.934	0.586
Circulatory failure	73.5% (100)	74.3% (26)	73.3% (74)		
Circulatory failure combined with respiratory failure	8.1% (11)	5.7% (2)	8.9% (9)		
Respiratory failure	9.6% (13)	14.3% (5)	7.9% (8)		
ECPR	8.8% (12)	5.7% (2)	9.9% (10)		
ECMO establishment sites				2.472	0.48
Operating room	26.5% (36)	31.4% (11)	24.8% (25)		
Monitoring room	58.8% (80)	60% (21)	58.4% (59)		
Emergency rescue room	1.5% (2)	0% (0)	2% (2)		
Catheter room	13.2% (18)	8.6% (3)	14.9% (15)		
Awake ECMO	17.7% (22)	9.4% (3)	20.7% (19)	2.069	0.15
With IABP	20.6% (28)	25.7% (9)	18.8% (19)	0.757	0.384

ECMO=extracorporeal membrane oxygenation; ECPR=external cardiopulmonary resuscitation; IABP=intra-aortic balloon pump

### Extracorporeal Membrane Oxygenation Complications

On comparing ECMO-related complications, the incidence of neurological complications was found to be significantly higher in the death group (22.9%) than in the survival group (5.9%) (*P*=0.012). The incidence of renal failure (assessed via dialysis) was significantly higher (42.9%) in the death group than in the survival group (19.8%) (*P*=0.007), as were the incidence of limb ischemia (40.0% *vs.* 17.0%) (*P*=0.005) and the incidence of infection (54.3% *vs.* 20.0%), with the latter being statistically significant (*P*<0.001) (Table 3).

**Table 3:** Comparison of ECMO complications.

	Total (n = 136)	Death group (n = 35)	Survival group (n = 101)	χ2	*P*-value
Bleeding	36% (49)	45.7% (16)	32.7% (33)	1.918	0.166
Neurological complications	10.3% (14)	22.9% (8)	5.9% (6)	6.327	0.012
Using CRRT	25.7% (35)	42.9% (15)	19.8% (20)	7.229	0.007
Limbs ischaemia	23% (31)	40% (14)	17% (17)	7.753	0.005
Infection	28.9% (39)	54.3% (19)	19.820% (20)	14.835	< 0.001

CRRT=continuous renal replacement therapy; ECMO=extracorporeal membrane oxygenation

### Binary Logistic Regression Analysis

Risk factors associated with the prognosis were analysed via binary logistic regression analysis. Here, in-hospital death was used as the dependent variable. The regression analysis was performed using all statistically significant risk factors under univariate analysis (age, Lac 24 hours after ECMO, in-hospital neurological complications, dialysis, continuous renal replacement therapy, limb ischemia, and infection) as the independent variables. The analysis results indicated that hospitalisation time (odds ratio [OR]: 1.051 [1.013-1.091], *P*=0.009) and infection (OR: 0.341 [0.125-0.930], *P*=0.036) affected the prognosis of the patients who survived 24 hours after undergoing VA-ECMO and withdrawal. Both factors were associated with the risk of death in the patients who survived 24 hours after receiving the treatment.

## DISCUSSION

In this study, the risk factors associated with 24-hour death in patients with circulatory failure receiving VA-ECMO were explored. The main findings can be summarised as follows: 1) the patients who died within 24 hours were older, had a higher incidence of Lac following ECMO treatment, and had a shorter hospitalisation time than the patients who survived; 2) the death group had a higher rate of complications than the survival group, including neurological complications, renal failure, limb complications, and infection; 3) in the patients who survived and were discharged, infection was associated with increased hospital stays and costs. The results also revealed several risk factors associated with a short-term prognosis in a subset of severe patients undergoing VA-ECMO, highlighting the importance of precise treatment in clinical practice.

As an effective supportive treatment for cardiopulmonary failure, ECMO has received an increasing amount of attention from clinicians. Many scholars are committed to identify the risk factors associated with the failure of ECMO treatment. Multiple factors were identified as predictors of death, including the female gender^[12]^, an advanced age, preoperative myocardial infarction, diabetes^[13]^, history of CPR prior to ECMO treatment, renal failure^[12]^, myocardial enzyme^[14]^, and time of mechanical ventilation^[15]^. Our results suggested that in-hospital death during ECMO was associated with age, carrying ECMO for transport, and Lac value following ECMO treatment for 24 hours, while it was not associated with concomitant diabetes, hypertension, history of heart failure, cerebrovascular accidents, and pre-ECMO CPR. The duration of shock progression in inter-hospital transferred ECMO-supported patients may affect their survival due to the waiting time for the ECMO team to arrive and the possibility of organ dysfunction.

The experienced ECMO centre has strict simulation training drills and practical experience for different categories of ECMO indications, meaning there was no statistical difference between the two groups in this regard. The VA-ECMO-related work is not limited to the monitoring ward and the operating room, with our ECMO team completing the consultation and cooperative treatment mode for severe patients in all areas of the hospital.

According to the Extracorporeal Life Support Organization’s (ELSO) extracorporeal CPR guidelines, ECMO-related complications can be divided into two categories: bodily complications and mechanical complications^[16]^. Among the bodily complications, bleeding is the most common. Of the 136 cases in this study, 49 cases involved bleeding and blood exudation, with an incidence rate of 36%, a similar rate to ELSO’s *in vitro* tissue statistics. While there was a high incidence of bleeding, we found no bleeding associated with hospital death. Bleeding sites were mainly the surgical incision site and the canulation site, with the main causes being incomplete haemostasis at the canulation or surgical site, lack of coagulation factors, thrombocytopenia and reduced function caused by heparin anticoagulant, and prolonged ECMO. We believe that if the patient has no active bleeding, the activated clotting time can be maintained at 160-180 seconds and reduced to 140-160 seconds in the presence of blood exudation, while attention should be paid to maintaining a certain flow rate. Blood routine tests were conducted daily, and the platelet counts were maintained > 50 × 109/L. Deficient coagulation factors should be supplemented if necessary. When the abovementioned measures are proved to be ineffective, surgery should be actively conducted.

Infection is a common complication in ECMO-supported patients, and this will prolong the hospital stay and increase the treatment costs^[17,18]^. Prevention of infection is always an important issue during ECMO treatment, and ECMO-supported patients are prone to secondary fungal infection after long-term use of wide-spectrum antibiotics, which has a poor prognosis and should be given adequate attention^[19,20]^. Of the 136 patients in this study, 39 suffered from infection, accounting for 28.9% of the total. The higher incidence of infection during ECMO is mainly associated with excessive surgical trauma and prolonged canulation, with these factors being the main reasons for the high occurrence of haematological infection. There was no statistically significant difference in infection between the survival group and the non-surviving group, but the OR for infection in the binary logistic regression analysis was 0.341. This difference in statistical results was likely due to the fact that some of the ECMO cases in the non-survivor group at our institution were ultimately classified as non-survivor cases because the patients’ families withdrew them from the treatment due to financial difficulties. This could be the possible reason why infection was not a risk factor associated with death in the statistical analysis of this study.

Renal insufficiency is also one of the most common complications of ECMO, ranking third in bodily complications^[21,22]^. Thirty-five of the 136 patients developed renal insufficiency, with an incidence rate of 25.7%. Statistical analysis revealed that renal failure was associated with nosocomial death, which was consistent with the findings of a previous study^[12]^. Therefore, great attention should be paid to kidney protection during ECMO supporting treatment.

The incidence of vascular complications in femoral VA-ECMO has been reported to range from 10% to 70%^[23^,^24]^. Azis et al.^[25]^ reported vascular complications in 18 (17.8%) of their 101 patients, with almost all requiring surgical intervention. While the risk of mortality was not elevated in these patients, an association between limb vascular ischaemia and mortality was observed in the current study (Table 4).

**Table 4:** Binary logistic regression analysis for ECMO patients.

	*P*-value	Odds ratio	95% confidence interval
Age	0.214	0.981	0.951~1.011
Hospital stay	0.009	1.051	1.013~1.091
Neurological complications	0.332	0.485	0.112~2.092
Using CRRT	0.456	0.664	0.226~1.949
Limbs ischaemia	0.401	0.610	0.193~1.932
Infection	0.036	0.341	0.125~0.930
Lac 24 h after ECMO	0.017	0.961	0.929~0.993

CRRT=continuous renal replacement therapy; ECMO=extracorporeal membrane oxygenation; Lac=blood lactate

### Limitations

This study demonstrated real-world evidence of VA-ECMO use in tertiary hospitals; however, the study has a number of limitations. First, this is a retrospective study that involved a small sample size and insufficient data records. Specifically, there were several non-surviving patients at the centre who did not continue the treatment and were discharged for financial reasons, meaning certain important variables could not be more fully described, which could have led to some bias in the results. Furthermore, given that this is a single-centre study, the generalisability of the findings is limited. In addition, the causes of death were not systematically collected, meaning the association between the complications and death could not be fully assessed.

## CONCLUSION

Various VA-ECMO-related complications, including kidney failure and infection, are associated with 24-hour in-hospital death. These risk factors should be given appropriate attention to improve the prognosis of patients undergoing VA-ECMO treatment.

## Abbreviations, Acronyms & Symbols

BMI: Body mass index
CPR: Cardiopulmonary resuscitation
CRRT: Continuous renal replacement therapy
ECMO: Extracorporeal membrane oxygenation
ECPR: External cardiopulmonary resuscitation
ELSO: Extracorporeal Life Support Organization
IABP: Intra-aortic balloon pump
ICU: Intensive care unit
Lac: Blood lactate
OR: Odds ratio
VA-ECMO: Venoarterial extracorporeal membrane oxygenation
